# Comparing the Emotion Status of Individuals With MCI to Their Cognitively Normal Counterparts: I-CONECT Project

**DOI:** 10.1093/geroni/igab046.1080

**Published:** 2021-12-17

**Authors:** Kexin Yu, Katherine Wild, Lisa Silbert, Jeffrey Kaye, Hiroko Dodge

**Affiliations:** 1 USC, LA, California, United States; 2 Oregon Health & Science University, Portland, Oregon, United States

## Abstract

Socially isolated older adults with MCI are at greater risk of developing ADRD. This study compares the emotional status of older adults with MCI to their cognitively normal counterparts within a socially isolated sample. We used baseline data from the Internet-based Conversational Engagement Clinical Trial (NCT02871921). MCI status was determined according to clinical diagnosis. Three emotion domain scores were calculated: negative affect, social satisfaction, and psychological wellbeing. Linear regressions were conducted for all 17 Emotion Battery measures and 3 domain scores. The 127 participants' mean age was 81.1 (SD=4.6). About 54% were diagnosed with MCI. Older adults with MCI had more negative affect, yet no difference was observed in social satisfaction and psychological wellbeing. Individuals with MCI had higher levels of fear, perceived hostility, perceived stress, sadness, and lower self-efficacy. Better understanding the emotional status could inform the development of behavioral health interventions and early detection of MCI.

